# The Foreign Journals: Anatomy and Physiology

**Published:** 1840-10

**Authors:** 


### PART THIRD.

### Selections from the British and Foreign Journals.

### I. THE FOREIGN JOURNALS.

### ANATOMY AND PHYSIOLOGY.

*On the Contractility of the Blood-vessels.* By Dr. Henle, of Berlin.

The object of the author in the present paper is to offer anatomical evidence in favour of the views respecting the nature of inflammation which he expressed in his "Pathological Investigations," and of which we gave a full analysis in our Number for April last. He now, in support of those opinions, advances that the structure of the middle coat of the artery is intermediate between cellular and muscular tissue; and that the vessels must therefore be liable to those changes of caliber under the direct or sympathetic influence of the nerves to which he believes that inflammation is due. With reference to his conclusions we can only repeat the opinion given in the review already mentioned, that mere dilatation of blood-vessels is not inflammation, and that even if it were proved that depression of the supposed motor nerves of the blood-vessels could result as an antagonist phenomenon of the excitement of the sensitive nerves, this would constitute but one and not the most important part of inflammation. However, the present anatomical observations of the author are not much the less interesting because he makes them the groundwork of incorrect pathology; if true, they afford additional proof, and indeed the only evidence that was wanting, to establish that the blood-vessels are subject to changes of their caliber under other than mere physical influences. And it is but fair that this evidence should be supplied from the school of Müller, who has done so much to throw discredit on the opinions of those who regarded the fact as already reasonably demonstrated.

The microscopic elements of the middle coat of the artery are broad and very flat slightly granulated fibres or bands, which lie in rings around the internal membrane. In the innermost layers they readily break up into tolerably similar long rhombic lamellæ, which are much like flattened cuticle cells, but are longer in proportion to their breadth. Of these lamellæ some are quite homogeneous, others present at one part a dark oval spot of the form of the common nuclei of cells which lies with its greatest diameter in the long axis of the lamella; and, lastly, on others this spot is drawn out into a longer and fine dark streak which often extends over the whole lamella but often over only a part of it, and often is replaced by a row of small dark points.

In the more external layers the lamellæ unite into long, and very seldom if ever branched fibres; the dark streaks also become connected together, not only joining with one another by their ends, but at the same time sending off lateral branches by which they anastomose. Thus the middle coat of the artery consists of numerous layers of granulated transverse bands (of 0·003 lines diameter), and of a system or network of dark streaks among them. These have some resemblance to elastic fibres, and have given rise to the opinion that this coat consists of elastic tissue. But a true elastic coat exists only on the larger arteries external to the middle one. The granulated bands of the middle coat dissolve in acetic acid, the dark branched streaks do not, and they may therefore by its means be easily demonstrated.

In the larger veins there is an exactly similar layer of transverse fibres next to the inner coat; but it is always very thin and may be entirely absent. (In the inner coat of the veins, however, a longitudinal layer of such fibres, which is much thinner or absent in the arteries, is in general considerably developed.) If one follows these fibres outwards it is seen how, though at first they were straight and stiff, they gradually assume the peculiar waviness of fasciculi of cellular tissue, and how at last there appears in them an obscure and then a more and more evident breaking up into separate parallel and tortuous filaments; for each granulated flat fibre there is a fasciculus of cellular tissue. The dark streaks on these fasciculi at first form a network like that which they present on the granulated fibres of the middle arterial coat; gradually they become finer and more translucent, the lateral branches fade, and nothing is seen but dark and very large wavy fibres, such as universally occur between the fasciculi of cellular tissue. These fasciculi become pale and dissolve in acetic acid; the fibres remain.

If the muscular coat of the stomach or of the intestine be examined successively from the serous to the mucous membrane, there are found externally lamellæ similar to those in the innermost layers of the middle arterial coat, with the same nuclei and the same development of the nuclei into streaks. Towards the mucous membrane the laminæ coalesce into broad fibres, the well-known organic muscular fibres which in their microscopic and chemical characters agree with the fibres of the middle coat of the artery, but which frequently exhibit obscure divisions into finer parallel fibres. The dark streaks are much finer, fewer, less branched, and more similar to the fine interstitial fibres of the cellular fasciculi just mentioned. The so-called muscular *fibres* of the stomach and intestines then correspond to the *fasciculi* of the cellular tissue and of the striated muscles.

I have not yet, says Dr. Henle, traced the transition from the organic muscular fibres to the striated muscular fasciculi, and I shall merely mention that in the latter also the system of dark interstitial fibres insoluble in acetic acid occurs sometimes in the form of nuclei, sometimes of longer streaks, and sometimes of extremely fine wavy fibres. It is well known that the organic and striated muscular fasciculi dissolve in acetic acid. The chemical difference between the middle coat of the artery and the muscles is explained by the predominant quantity of interstitial fibres in the former. The anatomical identity of the above-mentioned tissues in all essential points is thus, I believe, demonstrated: with the less essential anatomical differences there correspond differences of functions. The striated muscles are generally subject to the will and react on mechanical and electric stimuli, but not on the application of cold; the heart is an exception, having striated fasciculi but not being voluntarily moveable; the organic muscles react as the striated do, but are removed from the influence of the will; the middle coat of the artery is anatomically similar to the organic muscular membrane, it is, like them, involuntary, it reacts, like them, on mechanical stimuli, and also on the application of cold, but not of electricity; the contractile cellular tissues, for example, the tunica dartos, similarly involuntary, has the same relations to cold as the middle coat of the artery, but is not excited to contraction by mechanical impressions. In the animal muscles contraction following upon irritation is sudden and single; in the heart sudden and peristaltic; in the intestine slow and peristaltic; in the arteries and the cellular tissue slow and continuous.

The contractility of the larger arteries is proved by experiment, and we may venture to ascribe it to the smaller ones as far as they agree in structure with the larger, that is, so far as the layer of circular fibres is continued. Now this may be recognized on vessels of from 0·015 to 0·02 of a line in diameter by rendering them transparent with acetic acid and examining them under the microscope. The interstitial dark streaks are then seen running straight or somewhat obliquely round the vessel, at distances from each other which are equal to the breadth of a granulated fibre, in single layers on the small and in numerous layers on the large vessels. Separate fibres may also be exhibited on arteries of this size by tearing them. In smaller arteries, however, this is not possible; but even on those of 0·007 of a line in diameter (including their walls) there may be seen over the epithelium a layer of transverse, oval, and in part very elongated nuclei, which may be recognized as the origins of the dark interstitial fibres of the middle coat, by the circumstance that there is a gradual transition to the form of the latter, and that they already lie at the same distance from one another. Between them, therefore, the same substance must exist as between the interstitial fibres of the middle coat of the larger arteries, though it is not yet arranged in separate circular fibres. In vessels of this degree of minuteness the distinctions between arteries and veins are no longer to be discerned. Capillaries of less than 0·007 diameter (the smallest are from 0·002 to 0·003) have indeed separate transverse oval nuclei, but they have no special middle coat. The smallest consist only of a thin quite structureless membrane, in which oval nuclei lie here and there in a longitudinal position: those that are something larger have an epithelium of nuclei within this structureless primary membrane......

For the opinion that the contraction of the membrane of the vessels, like that of the muscles, is under the influence of the nerves, I can adduce an anatomical fact. Purkinje has seen fine nervous twigs on the cerebral vessels of sheep; and Valentin both on them and on many other blood-vessels; and I have myself often observed nervous filaments presenting the character of the so-called organic fibres on small vessels such as one can see undissected with powerful lenses. On a vessel of the pia mater of one fifth of a line in diameter I saw such a nervous fasciculus (of 0·009 of a line in diameter) ascending to the upper wall of the vessel which was turned towards the eye, then turning round it to its posterior wall, and then continuing its course in the same direction. I have seen this winding of the nerves round the vessels only on very small portions of the latter, but I have observed it so often that I cannot regard it as an accident. In one case a smaller fasciculus was given off; it consisted of only three or four fibres and passed along the vessel.

*Casper's Wochenschrift. Mai* 23, 1840.

---

### *On the Alterations which Nervous Fibres undergo after Division.*
### By Professor Nasse, of Marburg.

The results of the lengthened investigations which form the subject of this paper may be briefly stated.

In frogs, the natural average thickness of the filaments of the ischiatic nerve is 0·000416 inch; the majority are much smaller: the filaments of the posterior tibial are of about the same size; those of the brachial rather less, viz., 0·0003835. The smallest size observed was 0·00027 or 0·00028, and this was so common that the author is inclined to believe it to be the true diameter of all filaments unaltered by external influences, or at least of one kind of them. The limits of size were 0·00027 and 0·00045.

The filaments above the division, after it has healed (and usually about five months after the operation), are almost constantly larger than those below it. The average increase of size is 0·00005 to 0·00006.

The average size of the filaments which are paralyzed by the division is but little different from that of health; many months after the operation they are found only a little smaller. Their decrease in size is greatest when the animal is generally much emaciated; and is greatest of all when the main artery of the limb is divided at the same time as the nerve.

After destroying the connexion of a nerve with the spinal cord, its filaments first lose their cylindrical appearance, and from wrinkling acquire a transversely striated appearance, so that they seem to be divided into a number of short pieces. Next, small fat globules form from the breaking-up of the medulla, and the filaments become darker and more opaque. Afterwards these fat globules unite into larger drops, and then the walls of the nervous tubuli gradually diminish in their size.

The filaments that form in the substance by which the divided extremities are united are rather smaller than the old ones, but in all respects similar to nervous fibres; their average diameter is 0·000374.

Similar observations made on rabbits had very nearly the same results; but it was remarked that as in warm-blooded animals generally the nutrition of all the tissues is more under the influence of the spinal cord than in the cold-blooded, so here the destruction of the divided nerves was much more rapid in rabbits than in frogs; and that although kept without food for five months in the most unfavorable season of the year, the latter gave evidence of much more energy of reparation than the former which were well fed.

Although there was no doubt of new nervous fibres being formed between the extremities of the divided nerves, yet Nasse never found the motion or sensation return in the paralyzed limbs, though he sometimes kept the animal for three quarters of a year. He imagines that in the cases in which others have seen such a restoration of power, the divided portions must have been at once placed in exact apposition, so that the filaments could not lose their oily contents; for he clearly determined that the least alteration in the structure of a filament is sufficient to destroy its conducting power.

*Müller's Archiv*, 1839. Heft v., p. 405.

---

*Experimental Researches on the Functions of the Encephalon, considered in relation to Sensation, Station, and Progression.* By M. Monat.

This is a long report to the Royal Academy, in which the author, after proving the insensibility of the brain, endeavours to point out the functions of its different portions. In this, however, he has but partially succeeded, and the Committee, MM. Ribes, Blandin, Amussat, and Bouillaud come to the following conclusions: 1, That the three great nervous centres, the spinal marrow, the cerebrum, and the cerebellum, have distinct and special functions; 2, that sensation, properly so called, and motion are under the immediate and direct influence of the spinal marrow, and have each particular nerves; 3, that the brain presides over the various manifestations of intelligence and volition; 4, that the cerebellum plays an important part over the functions of *Progression* and *Station.* As to the functions peculiar to particular parts of the nervous centres, much yet remains to be done for their determination.

*Bull. de l'Acad. Roy. de Méd. Juin* 17, 1840.

---

*Philosophical and Experimental Researches into the True and Primary Action of the Secale Cornutum as a Medicine.* By Dr. C. T. De Gravina.

The first experiments were made upon five full-grown healthy sparrows, to three of which eight whole grains of the ergot were given, and to the two others six grains a piece, powdered and formed into pills by means of syrup. The two latter were the first to feel the effects of the drug, and then those which had taken the whole grains; but both exhibited the same phenomena. They first ceased to chirp and flutter when the cage was approached; they then remained immovable and could with difficulty support themselves; next they erected their feathers, which they shook from time to time, as though to free themselves from something that oppressed them and irritated their skins; then they violently shook their heads from side to side, opening and shaking their beaks, with an effort to vomit, which after a time took place, when they threw up fragments of the pills or whole grains of ergot, soaked in a clear and frothy fluid like saliva. The muscular weakness increased to perfect immobility unless they were much teazed, when they removed a few steps from the spot and relapsed into their former state; and when thrown into the air they soon fell to the ground. The respiration and pulse became slower, and with the animal heat decreased gradually till their death, which took place in six or seven hours after swallowing the ergot. Examined immediately after death, the viscera presented no inflammatory morbid appearances. The lungs were healthy, a little congested. The right ventricle, auricle, and the venæ cavæ were distended with blood, and the spleen was much congested. The brain presented no morbid changes, but the muscles were fragile and contained black blood. Two guinea-pigs were the subjects of the next experiment, a male and a female, the latter ten days advanced in her second pregnancy. Twenty-four grains of ergot, powdered, and made into a paste with flour and water, were given to each daily before their other food, and the dose was increased each day by the addition of twelve grains till it amounted to seventy-two grains. In about an hour after each dose they became immovable and indifferent to any disturbance, but recomposed themselves immediately. The heart beat very slowly, and they refused every kind of food. Two or three hours were passed in this state, and then they returned to their former liveliness; but the effects of the ergot were more distinct and permanent in proportion as the dose was augmented. The female, though pregnant, exhibited no increase in size during the eight days that she took the ergot, nor indeed after it had ceased to be given; so that Dr. De Gravina began to doubt the fact of her pregnancy, especially as the time for her bringing forth had now passed; and although her belly might have been rather tumid yet that was attributed to fat, as she took a great deal of food, and had been confined from the commencement of the experiment in a pen by herself. However, twelve days after the usual period she produced three very lively young ones, one of which was blind of the right eye.

Wishing to try the effects of the ergot upon a healthy person, Dr. De Gravina swallowed twenty-four grains in powder, which in three quarters of an hour produced a sensation of weight in the epigastrium, which, slight at first, became in a short time very painful. This was followed by nausea and eructations having the odour of the ergot, and terminating in attempts to vomit. His face became very pale and the skin cold, particularly that of the face; there was great oppression in the head with incapacity for any mental exertion, apathy, and a feeling of complete prostration and vertigo on walking across the room. The pulse, which before the experiment was 65, fell to 54, small and slow; and the respirations decreased from 18 to 13 per minute. There was a complete disgust for food, with an occasional feeling of coldness at the stomach that thence pervaded the whole frame. All these symptoms were instantly removed and a voracious appetite was restored by simply taking a glass of strong wine.

Having thus, he says, ascertained that wine was an antidote to the effects of ergot, Dr. De Gravina resolved to try whether opium and mace would have an equal power to prevent the sedative action of the drug when administered with it; but, in order to learn what would be the effect of the opium and mace given separately, he previously instituted the following experiments:

A grain of opium was given to a sparrow just caught, and a grain and a half to another. In half an hour the former became exceedingly lively, chirping, fluttering, and turning his head in every direction in a most excited manner for twenty minutes, when he fell asleep. His breathing was frequent and his heart beat most rapidly, and the heat of his body was much increased. Roused by a kind of convulsion, he vomited a fluid like saliva in which a little opium was dissolved, and then recomposed himself to sleep, during which universal tetanic spasms came on, followed by paralysis with atonic spasms of the right side. The tetanus became more frequent and violent, and in one attack he vomited a larger quantity of similar matter as before; the attacks then became gradually less violent and frequent; he fell again into a deep sleep, from which awaking by degrees he returned to his previous state of health. The second sparrow exhibited the same symptoms but in a more violent degree, and having been prevented from vomiting by a ligature round the neck, the tetanic spasms continued incessantly to his death. The cerebro-spinal axis was vividly injected in every part, and the digestive tube was much the same; the lungs were gorged with florid blood, and the muscles were rigid and tore with difficulty, nor were they at all injected with dark blood as in the experiments with the ergot.

Having now established (as he imagined) the action of both substances singly, Dr. De Gravina gave another sparrow half a grain of opium with three grains of ergot in a pill. In three quarters of an hour the effects of the opium appeared as before with great liveliness of motion, but instead of passing into a state of stupor, the animal became depressed and exhibited all the signs of poisoning by ergot. In this state he gave it four drops of laudanum, which instantly removed all the symptoms. In repetitions of the last experiment, the opium always counteracted the effects of the ergot, with or without exhibiting its own, according to the proportion it bore to the dose of ergot.

The effects of mace were much the same as the early symptoms produced by opium, but in an extreme degree; and after the animals had remained about half an hour in this state, a slight opisthotonos appeared, increasing rapidly till near the death of the birds, when they began a progressive movement in a straight line, turning neither to left nor right, but continuing to push against any obstacle that opposed itself insurmountably to their progress, and moving their legs as though they were swimming. A strong bronchial rattle with panting came on before death, which occurred about four hours after they had taken the mace. Immediate dissection disclosed the intestinal tube highly injected; the lungs hard, hepatized, and engorged with very florid blood; a bloody serous fluid filled the trachea; the parietes of the heart were redder than usual; the venous blood was of a bright red and gave out a strong smell of nutmeg; the vessels of the brain and spinal cord were very conspicuous and of a most brilliant colour. These experiments were repeated with various doses of the mace, but with the same results; and also with mace combined with ergot, when each in turn produced its peculiar effects, but with a modified intensity, as resulted from the combination of opium and ergot.

Cicuta given to sparrows produced direct depression and stupor without any previous excitement, and combined with ergot, death without a struggle. The morbid appearances were the same as in poisoning with pure ergot.

Dr. De Gravina next cites a case of irritable uterus with diseased ovary, first relieved by cicuta, and in a second attack by ergot, to prove the identity in the mode of action of these remedies; and several cases of hæmoptysis and active inflammations treated with bleeding, ergot, ice, and digitalis, in order to establish the same point in regard to them.

From these experiments and cases, Dr. De Gravina concludes that ergot, like cicuta and digitalis, is a direct sedative, and that it is as directly opposed in its action to opium, mace, and wine. Hence he considers it a good antiphlogistic remedy, and well calculated to lower the vital powers.

With regard to its action on the uterus, he cites several cases from which he concludes that the condition of the uterus that renders it liable to be stimulated to contraction upon its contents, is when there is a degree of morbid vigour in the organ, and not when it is in a state of real debility, for then the ergot is worse than useless as it induces general depression.

[The reader will at once perceive the imperfections in the mode of experimenting, and the chasms and weak points in the chain of reasoning in the foregoing paper. Still the experiments have some value as single facts; and they deserve more consideration from their results coinciding with those of Mr. Wright, (Edin. Med. and Surg. Journal, No. 142,) more particularly in the fact of the power of ergot in prolonging gestation in the guinea-pig and rabbit, when administered for a considerable period.]

*Annali Universali di Medicina. Ottobre,* 1839.

